# Inferring the evolutionary mechanism of the chloroplast genome size by comparing whole-chloroplast genome sequences in seed plants

**DOI:** 10.1038/s41598-017-01518-5

**Published:** 2017-05-08

**Authors:** Zheng Xiao-Ming, Wang Junrui, Feng Li, Liu Sha, Pang Hongbo, Qi Lan, Li Jing, Sun Yan, Qiao Weihua, Zhang Lifang, Cheng Yunlian, Yang Qingwen

**Affiliations:** 1 National Key Facility for Crop Gene Resources and Genetic Improvement, Institute of Crop Sciences, Chinese Academy of Agricultural Sciences, Beijing, 100081 China; 2 College of Chemistry and Life Science, Shenyang Normal University, Shenyang, 110034 China

## Abstract

The chloroplast genome originated from photosynthetic organisms and has retained the core genes that mainly encode components of photosynthesis. However, the causes of variations in chloroplast genome size in seed plants have only been thoroughly analyzed within small subsets of spermatophytes. In this study, we conducted the first comparative analysis on a large scale to examine the relationship between sequence characteristics and genome size in 272 seed plants based on cross-species and phylogenetic signal analysis. Our results showed that inverted repeat regions, large or small single copies, intergenic regions, and gene number can be attributed to the variations in chloroplast genome size among closely related species. However, chloroplast gene length underwent evolution affecting chloroplast genome size in seed plants irrespective of whether phylogenetic information was incorporated. Among chloroplast genes, *atpA*, *accD* and *ycf1* account for 13% of the variation in genome size, and the average *Ka*/*Ks* values of homologous pairs of the three genes are larger than 1. The relationship between chloroplast genome size and gene length might be affected by selection during the evolution of spermatophytes. The variation in chloroplast genome size may influence energy generation and ecological strategy in seed plants.

## Introduction

The variation in genome size, which simultaneously reflects genotype and phenotype, has been a puzzle for researchers for almost half a century^1–3^. Previous studies have reported the significant associations between the variation in genome size and life history^4, 5^, taxonomy^6^, evolutionary affiliation^7^ and geographical distribution^8^. These associations were suggested to be determined by selective force^1, 3, 9^. Genome size change has also been linked to remarkable changes in non-coding sequences, and random drift is regarded as a strong evolutionary force that affects genome size variation^10, 11^. However, these associations between DNA composition and genome size^2, 9^ have not been clarified in species over a broad range of evolutionary time. Currently, the development of genome sequence technology and population genetics methods has enabled researchers to identify the signatures of selection or genetic drift of genome size variation^12, 13^.

Chloroplasts originated from endosymbiotic photosynthetic organisms and retain their own unique DNA encoding multiple genes, including components of light reactions in the photosynthesis process to convert light energy into chemical energy^14, 15^, and photosynthesis is strictly controlled by the genes in chloroplasts^16^. Most plant chloroplast genomes have been examined, and they have a very constrained size that ranges from 120 kb to 160 kb^17^. The limited size change in chloroplast genomes in nearly all of the main lineages in plants indicates the possibility that the chloroplast genome is maintained by natural selection, especially when compared to the random and large-scale size variations in both mitochondrial^18^ and nuclear genomes^19^. In seed plants, the chloroplast genome exhibits a conserved genome structure^17^ that includes two inverted repeats (IRs), through which a long single-copy section (LSC) and a short single-copy section (SSC) are separated. In addition, compared to nuclear and certain plant mitochondrial genomes, chloroplast genomes are small and less prone to recombination, which provides distinct information for studying genome size variation and evolutionary status^20, 21^. These described features are advantageous for comparative studies because they enable researchers to investigate genome divergences over a broad range of evolutionary time, from early land plants^22^ to recently domesticated plants, and to detect selection signals of genome size evolution^23^.

Three important factors have been proposed to drive the variation in chloroplast genome size in previous studies of seed plants: (a) intergenic region variation, which mainly affects the variation in chloroplast genome size within a genus^24–27^; (b) variation of an IR region, which is an important characteristic of specific groups, such as gymnosperms, Poaceae and Leguminosae^28–35^; and (c) gene loss, which is an important reason for the shrinking of chloroplast genome size in some parasitic plants^28, 35^. However, previous studies of chloroplast genome size that have used limited taxon sampling or comparisons among very distant relatives have yielded results of uncertain generality, and there is a lack of systemic and comprehensive phylogenetic studies. It remains unclear which of the three factors has a greater influence on genome size, and the recontribution of natural selection to genome variation is still unknown.

In this study, we collected and annotated 272 complete chloroplast genomes of seed plants, and phylogenies were constructed as a basis to infer the evolutionary mechanism of chloroplast genome size. We first analyzed the general structures of the 272 chloroplast genomes with phylogenetic information incorporated; then, we compared the general structures of chloroplast genomes among monocots, eudicots, basal angiosperms and gymnosperms. Second, we assessed the influence of different sequence characteristics on the variations in chloroplast genome size through a conventional analysis of variance based on cross-species and phylogenetic signal analyses. The analyses suggested that variations in genome size originate from lineage-specific differences in intergenic region variation, and the generality of the genome size and gene length relationship was confirmed by a cross-species and independent contrasts analysis. Finally, the variation patterns and the results of principal component analyses of 126 chloroplast genes were compared among the 272 species. It was demonstrated that *atpA*, *accD* and *ycf1* may influence photosynthesis for plant adaptation. The variations in chloroplast genome size may play an important genetic role in influencing the energy generation and ecological strategy of a species.

## Results

### General variation of the chloroplast genome in seed plants

**Figure 1:**
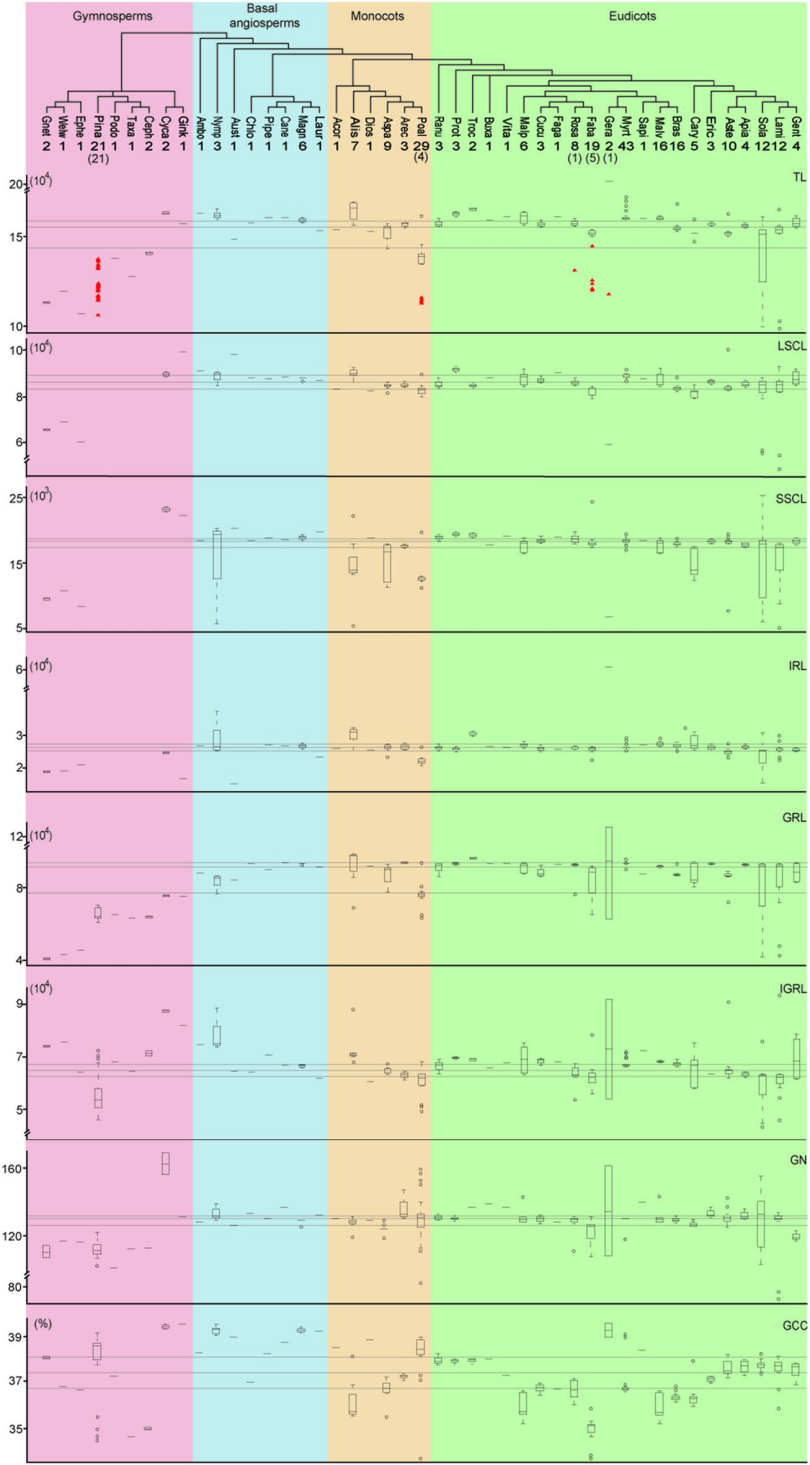
Variations in chloroplast genome size and the sequence characteristics of chloroplast genomes within seed plants. The box plots of chloroplast genome size and sequence characteristics of chloroplast genome are shown for each order. The complete order names are in Supplementary Table S1. The box plots represent the median (central line), first and third quartiles (black box), and outliers (black circles). TL, IRL, LSCL, SSCL, GRL, IGRL, and GN indicate the total length of the chloroplast genome, length of the inverted repeat region, large single copy, small single copy, gene region, intergenic region, and gene number, respectively. Red triangles in TL indicate there were no inverted repeat regions in these species. The numbers below order names are the number of species collected in each order. The number in the brackets indicates the number of species without an inverted repeat region. The three lines from the top to bottom represent the first quartiles, the median, and the third quartiles of the sequence characteristics of all seed plants examined in this study, respectively.

Two-hundred and seventy-two complete or nearly complete chloroplast genomes were collected from 67 families of 45 orders, including 32 genomes from gymnosperms, 15 from basal angiosperms, 50 from monocots and 175 from eudicots respectively. The 32 gymnosperm chloroplast genomes were derived from 10 families, which included all the major basal lineages of gymnosperms except Araucariaceae and Taxodiaceae. The 240 angiosperm chloroplast genomes were collected from 57 families, including all eight orders of basal angiosperms, six (out of 12) orders of monocots and 22 (out of 43) orders of eudicots, which covered the four major branches fabids, malvids, lamiids and campanulids. General variations in the chloroplast genome were analyzed in these species for the total length of the chloroplast genome (TL), the length of the inverted repeat region (IRL), large single copy (LSCL), small single copy (SSCL), gene region (GRL), intergenic region (IGRL), GC content (GCC), and gene number (GN) (see Supplementary Table S1 and Fig. 1).

The TL ranged from 70,028 bp (*Epifagus virginiana*, eudicot) to 217,942 bp (*Pelargonium x hortorum*, eudicot). The median and the first and the third quartiles of TL were 155,621 bp (*Fragaria virginiana*, eudicot), 160,076 bp (*Eucalyptus marginata*, eudicot) and 143,164 bp (*Erycina pusilla*, monocot), respectively. *Trachelium caeruleum* (eudicot), *Capsicum annuum* (eudicot) and *Pelargonium x hortorum* (eudicot) had the longest LSCL (100,114), SSCL (25,783) and IRL (75,741), respectively. The LSCL (19,799) and SSCL (4759) of *Epifagus virginiana* (eudicot) were smaller than those of other species, and the IRL (15,114) of *Illicium oligandrum* (basal angiosperms) was the smallest among all the existing chloroplast genome sequences. The coefficients of variation (standard deviation/mean) of SSCL (0.20) and IRL (0.17) were nearly twice as high as those of LSCL (0.08), IGRL (0.11) and GRL (0.10), which indicated that more samples deviated from the average in the distributions of SSCL and IRL than in those of LSCL, IGRL and GRL. The coefficient of variation of TL (0.09) was close to that of LSCL and GRL. The variation of GCC in chloroplast genomes was small and ranged from 33.80% (*Typha latifolia*, monocot) to 39.60% (*Pelargonium x hortorum*, eudicot). The coefficient of variation for GCC was only 0.03. The GNs ranged from 56 (*Epifagus virginiana*, eudicot) to 165 (*Oryza nivara*, monocot), and the GNs of 87% species ranged from 110 to 140. The coefficient of variation of GN (0.09) was the same as TL.

**Figure 2:**
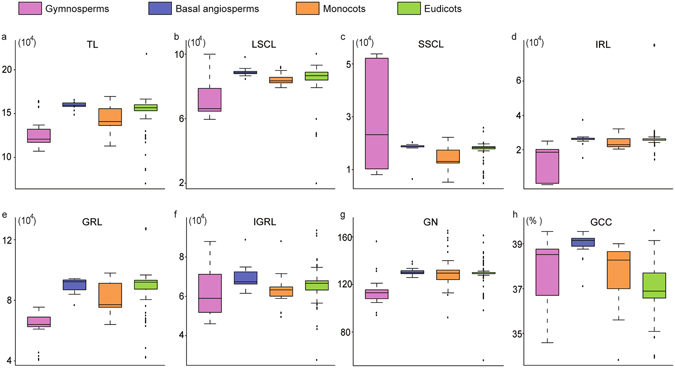
Variations in chloroplast genome size and the sequence characteristics of chloroplast genome in gymnosperms (pink), basal angiosperms (blue), monocots (yellow), and eudicots (green). The box plots represent the median (central line), first and third quartiles (black box), and outliers (black circles).

We further compared the general variations in chloroplast genomes among monocots, eudicots, basal angiosperms and gymnosperms using a *t*-test (Fig. 2). LSCL and GRL showed a similar distribution to TL in gymnosperms, basal angiosperms, monocots, and eudicots. The median values of TL (*p* = 0.001), LSCL (*p* = 0.08) and GRL (*p* = 0.001) of gymnosperms were significantly lower than those of angiosperms according to a *t*-test. In angiosperms, the monocots had a significantly smaller chloroplast genome size compared to eudicots and basal angiosperms (*p* = 0.003 for TL; *p* = 0.001 for LSCL; *p* = 0.003 for GRL). Although the median values of TL, LSCL and GRL were similar in eudicots and basal angiosperms, basal angiosperms (13 for TL; 6 for LSCL; 13 for GRL) had more outliers than eudicots (5 for TL; 2 for LSCL; 1 for GRL). For SSCL and IRL, monocots, eudicots and basal angiosperms had similar ranges and variations (*p* = 0.23), but gymnosperms had higher ranges and variances than angiosperms (*p* = 0.003). The distribution of IGRL (*p* = 0.16) and GCC (*p* = 0.53) was not significantly different among monocots, eudicots, basal angiosperms and gymnosperms. The GN (*p* = 0.02) of gymnosperms was significantly smaller than that of angiosperms, whereas monocots, eudicots and basal angiosperms had similar median GN values (*p* = 0.51).

### Factors influencing chloroplast genome size based on cross-species and phylogenetic signal analyses

We found that inverted repeat region variation and gene loss occurred many times independently. To further explore the relationship between genome size and other characteristics (see Supplementary Table S1), a conventional analyses of variance (ANOVA) was performed based on cross-species and phylogenetic signal analysis (Table 1). For cross-species analyses, large amounts of data related to chloroplast genome size and chloroplast sequence characteristics among species were collected (Fig. 1), and analyses across all species showed that chloroplast genome size was significantly and positively associated with IRL, LSCL, SCCL, GRL, IGRL and GN but not GCC (Table 1). The variations in chloroplast genome size could explain the lower variations in IRL (*r* ^*2*^ = 23%), LSCL (*r* ^*2*^ = 24%) and SCCL (*r* ^*2*^ = 25%) than in GRL (*r* ^*2*^ = 81%), IGRL (*r* ^*2*^ = 69%) and GN (*r* ^*2*^ = 76%). The estimated slopes for chloroplast genome size and IRL (0.05), LSCL (0.04) or SCCL (0.05) were similar and were significantly smaller than those for GRL (1.11), IGRL (0.83) and GN (0.74) (Table 1).

**Table 1:** Standardized major axis (SMA) slope estimates describing the relationships between chloroplast genome size and TL, IRL, LSCL, SCCL, GRL, IGRL, GCC and GN for both cross-species and based on phylogenetic signal analyses.

	Chloroplast genome size						
	Cross-species			Phylogenetic			*K*
	*r* ^*2*^	Slope	95% CI	*r* ^*2*^	Slope	95% CI	
IRL	0.23	0.05	(0.04, 0.05)	0.11	0.03	(0.03, 0.04)	0.92
LSCL	0.24	0.04	(0.04, 0.05)	0.1	0.03	(0.03, 0.05)	0.93
SCCL	0.25	0.05	(0.04, 0.05)	0.11	0.04	(0.04, 0.05)	0.92
GRL	0.81	1.11	(1.07, 1.13)	*0*.*64*	*0*.*91*	(*0*.*84*, *0*.*98*)	0.59
IGRL	0.69	0.83	(0.78, 0.89)	0.12	0.03	(0.03, 0.04)	0.95
GCC	0.09	0.29	(0.25, 0.33)	0.08	0.19	(0.15, 0.22)	0.97
GN	0.76	0.74	(0.69, 0.76)	0.13	0.07	(0.06, 0.07)	0.96

Statistically significant values are indicated in italics.

TL, IRL, LSCL, SSCL, GRL, IGRL and GN indicates the total length of chloroplast genome, the length of inverted repeat region, large single copy, small single copy, gene region, intergenic region, and the gene number respectively. *K* describes the degree of the difference between the *F*-statistic of simulated data and observed *F*-statistic distributions.

The genome character and sequence variation are significantly associated with phylogenetic signal^36^. Therefore, closely related species are more likely to have similar genome sizes and characteristics. We first compared the data from the above comparative analysis with the values obtained from 1000 Monte Carlo simulations that randomized the data from the phylogeny tree. We used *K* to describe the degree of the difference between the *F*-statistic of simulated data and observed *F*-statistic distributions. The descriptive statistics (*K*) of LSCL (*K* = 0.93), IGRL (*K* = 0.95), GN (*K* = 0.96) and IR (*K* = 0.92) were close to 1 and larger than GRL (*K* = 0.59), indicating that LSCL, IGRL, GN and IR were more strongly affected by the phylogenetic signal than was GRL. Therefore, we performed a conventional analysis of variance with phylogeny being taken into account and compared the chloroplast genome size with genome characteristics standardized by branch lengths. The slope estimate between chloroplast genome size and GRL obtained from the comparison based on phylogeny analyses (0.91) was significantly smaller when compared to the cross-species results (slope = 1.11). However, the inclusion or exclusion of phylogeny in the analyses for chloroplast genome size and IRL, LSCL, SCCL, IGRL or GN did not lead to any differences in *r* ^*2*^ (Table 1).

IRL, LSCL, SCCL, GRL, IGRL and GN had the same distribution in four phylogenetic groups as TL (Fig. 2). However, the critical values (*C*) based on a phylogenetically corrected ANOVA, which were obtained from a distribution of 1000 Monte Carlo simulated *F*-statistics assuming a gradual model of Brownian motion, suggested that IRL (*C* = 70.52, *p* = 0.25), LSCL (*C* = 76.41, *p* = 0.35), SCCL (*C* = 71.46, *p* = 0.55), IGRL (*C* = 34.51, *p* = 0.15) and GN (*C* = 26.42, *p* = 0.07) were not significantly associated with chloroplast genome size. In other words, the associated relationship observed among IRL, LSCL, SCCL, IGRL and GN could be attributed to phylogenetic signaling. However, after incorporating both chance and phylogeny into the ANOVA, the variations in chloroplast genome size were significantly associated with the variations in GRL (*C* = 18.26, *p* = 0.00002), which were higher than the values predicted by the Brownian motion model. The above results indicate that the variation in gene length plays an important role in the variation in chloroplast genome size.

### Comparison of chloroplast genes among seed plants

So we explored the variation patterns of chloroplast genes and we standardized gene annotation and gene length. All collected chloroplast genomes were re-annotated using DOGMA with default settings. Two or more successive genes with the same name were annotated as one gene, such as *clpP* and *rpl2*, which had more than one exon. Multiple genes with overlapping regions were manually adjusted into one gene, such as *orf188* and *ndhA*. The genes annotated in only one species, including *orf221*, *orf332*, *orf365* and *orf574*, were not used in subsequent analyses. In addition, we standardized gene length based on its average and variance. We obtained standardized contrasts (SCs) for further analyses by dividing the difference between the gene length of each species and the average total gene length by the standard deviations.

A total of 126 chloroplast genes were annotated, which were divided into three broad categories and 13 subcategories. The first category (I) comprised genes for the photosynthetic apparatus, including 6 photosystem I, 15 photosystem II, 7 cytochrome b6f, 6 ATP synthase, 1 RuBisCo and 11 NAD(P)H dehydrogenase genes; the second category (II) comprised RNA genes and genes for the genetic apparatus, including 31 transfer RNA, 4 ribosomal RNA, 4 RNA polymerase and 21 ribosomal subunit genes; and the third category (III) consisted of potential genes, including 8 conserved hypothetical chloroplast open reading frames (ycfs), 2 open reading frames (ORFs) and 10 potential protein-coding genes. A total of 106 genes (81% of the total) were found in more than 90% (245) of the species, and 13 genes were found in less than 10% of the species (27). Among these low-frequency genes, three were photosynthetic apparatus genes, one was a tRNA gene and nine were ORFs or ycfs. All of these rare genes were found in gymnosperms and a small proportion of angiosperms (such as Fabaceae, Cucurbitaceae, Araceae and Geraniaceae).

**Figure 3:**
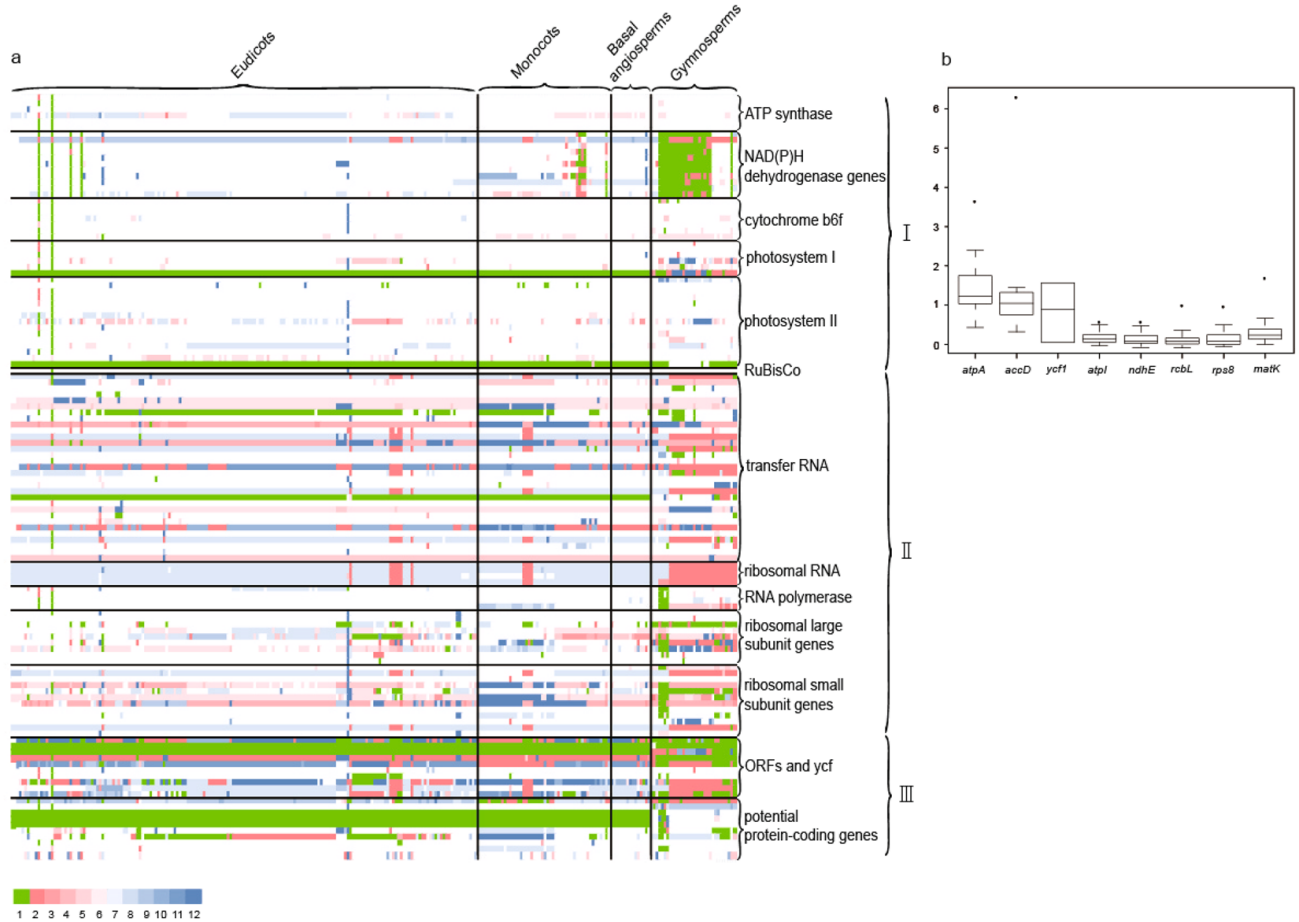
Relationship between chloroplast genome size and chloroplast gene length. (**a**) Heat maps of standardized contrast (SC) values for each gene. SCs were obtained by dividing the raw contrasts between gene length and the average gene length by the standard deviation. Green indicates that the gene was lost in the species. White indicates that SC is zero, which indicates that the sequence characteristics of this species were equal to the average of the sequence characteristics of all seed plants collected for this study. Red stands for an SC larger than zero, blue stands for an SC smaller than zero, and larger absolute values of SC are indicated by darker colors. I indicates genes for the photosynthetic apparatus, II comprises RNA genes and genes for the genetic apparatus, and III represents potential genes. (**b**) The box plots of *Ka*/*Ks* values of *atpA*, *accD*, *ycf1 atpI*, *ndhE*, *rbcL*, *rps8*, and *matK* in the homologous genes of plants.

The SC of each gene is shown in Fig. 3a. The coefficient of variation for the SC of each gene ranged from 0.052 (*trns*-UGA) to 16.49 (*orf574*) in all genes, and the average was 1.02. The variation in SC of the genes in the first category was smaller than that of the genes in the second and third categories. Principal component analysis of SC indicated that *atpA*, *accD* and *ycf1* accounted for 13% of the variation in plant chloroplast genome size. The gene *atpA*, which codes for a small photosystem II polypeptide, *accD*, which affects leaf development, and *ycf1*, which is associated with plant survival^37^, may influence photosynthesis and are associated with plant adaptation. Therefore, variations in these genes during plant evolution may play an important genetic role in determining the energy generation and ecological strategy of a species. Thus, we detected the selection signal of these genes by calculating the *Ka*/*Ks* of their homologous gene pairs with the most recent common ancestor of the plants, and the SC of these gene pairs was larger than 1. In addition, we selected five genes (*atpI*, *ndhE*, *rbcL*, *rps8*, and *matK*) that had smaller effects on the length of genome than *atpA*, *accD* and *ycf1* according to principal component analysis. Strength of selection is commonly measured by calculating the ratio of nonsynonymous (change in amino acid) substitution over synonymous (silent) substitutions (*Ka*/*Ks*). We calculated the *Ka*/*Ks* values associated with terminal branches to measure the strength of selection during the most recent divergence of each species. We investigated the *Ka*/*Ks* values for the 8 protein-coding genes from the chloroplast genome, including *atpA*, *accD*, *ycf1*, *atpI*, *ndhE*, *rbcL*, *rps8*, and *matK* (Fig. 3b). The average *Ka*/*Ks* values of *atpA*, *accD* and *ycf1* in homologous genes of plants were greater than 1, which was larger than that of other genes.

## Discussion

The main purpose of this study was to re-examine the relationship between chloroplast genome size and chloroplast sequence characteristics within seed plants using large datasets of species and two comparative approaches. Across 272 species of 67 families, we found that the variations in the chloroplast genome in closely related species were affected by intergenic region length. The log-scale linearity in the relationship between chloroplast genome size and chloroplast gene length was revealed by both cross-species and phylogenic analyses (Table 1). Moreover, we found that across all species, IGRL or GN accounted for more than 60% of the total variation in chloroplast genome size. However, tests of phylogenetic signal indicated that this pattern of GN was not independent of ancestry. The variations in IRL, LSCL and SCCL were related to the variation in genome size, but they only explained nearly 20% of the total variation in chloroplast genome size based on cross-species analyses (Table 1). Therefore, our results support the assumption that chloroplast gene length is a predictor of plant chloroplast genome size across a long evolutionary timespan (Fig. 3a and Table 1). Additional factors, such as IGRL, GN, IRL, LSCL and SCCL, may play a role in determining variations in the chloroplast genome among closely related species (Table 1), and IRL, LSCL and SCCL may only modulate the chloroplast genome size of a few groups of species (Fig. 1). The variations in IGRL, GN, IRL, LSCL and SCCL are an important reason for variations in chloroplast genomes in closely related species.

Previous studies have reported that gene loss^28, 35^, inverted repeat region variation^29, 32–34^ and intergenic region variation^24–27^ are three important factors driving the variation in chloroplast genome size in plants. Chloroplast gene loss is an important reason for the reduction of chloroplast genomes in some parasitic plants^38–42^. These parasitic plants are not closely related in phylogeny, but all have undergone similar functional changes in photosynthesis^40–42^. However, both intergenic region variation and inverted repeat region variation associated with the chloroplast genome length diversity were observed in previous study based on comparisons within a genus or a family. For example, the loss of the inverted repeat region often occurs in some species groups, such as Fabaceae^43, 44^, Pinaceae^45^ and Geraniaceae^46^, and this loss is very rare in most other plant families (Fig. 1). Intergenic region variation was the result of comparisons of chloroplast genome length among species within a genus, such as Poaceae^10^ and Orchidaceae^47, 48^. All these previous research studies also supported our conclusion.

Chloroplast gene length is an important factor affecting the variations in chloroplast genome size based on phylogenetic signals (Table 1). This result contrasts with the results for nuclear genome size, which was primarily affected by the non-protein-encoding fraction of the genome^49–53^. Three reasons may explain this outcome: (1) the mutation and recombination rate of the chloroplast genome is much lower compared to the nuclear genome, which results in fewer repeat sequences and transposons^54, 55^; (2) the chloroplast genome originated from endosymbiotic photosynthetic organisms and retained core genes, which led the length of gene region of the chloroplast genome to be significantly larger than the intergenic region in most plants^56–58^; and (3) the chloroplast genome originated from prokaryotes, whose GN and genome size were strongly correlated because prokaryotes generally exhibit a paucity of non-coding DNA^59–61^.

In our study we considered the phylogenetic factors, which is a big step forward. Our results demonstrate that chloroplast gene length is significantly associated with chloroplast genome size based on both cross-species and phylogenic signal analyses across 272 species. Moreover, we found that among all chloroplast genes, *atpA*, *accD* and *ycf1* accounted for 13% of the variation in plant chloroplast genome size through principal component analysis (Fig. 3b). *AtpA*, *accD* and *ycf1* may influence photosynthesis and may be useful for predicting plant responses to environment variation^62–65^. *AtpA*, *accD* and *ycf1* have been completely or partially lost in the plastid genome multiple times during evolution. In the grass chloroplast genome, the degradation of *accD* and *ycf1* occurred in the ancestors of grass. In addition, the *accD* reading frame underwent a length expansion in cupressophytes^66^. We also found that the average *Ka*/*Ks* values of the homologous gene pairs of the three genes in plants were higher than 1 (Fig. 3b). These findings indicate that these genes, which have a considerable effect on the variations in chloroplast genome size, have undergone strong selection^67^. Generally, nonsynonymous substitutions are more likely to cause functional changes than synonymous substitutions because the mutation accumulation of *atpA*, *accD* and *ycf1* can cause variations in photosynthesis efficiency. The relationship between chloroplast genome size and functional gene content variation suggested that the variation in chloroplast genome size may influence photosynthesis, which may cause a higher level of ecological diversity for organisms. These results are important for understanding the processes underlying the complexity of chloroplast genomes and highlight the interdependence between chloroplast genome size and environmental complexity.

## Materials and Methods

### Plant materials and genome annotation

A total of 272 complete or nearly complete chloroplast genomes were collected from NCBI (National Center for Biotechnology Information), including the genomes of five gymnosperm groups, four clades of eudicots (fabids, malvids, lamiids, and campanulids), one major clade of monocots (commelinids), and basal angiosperms (magnoliids). The details (species name, family names, and accession numbers) of 272 chloroplast genomes are listed in Supplementary Table S1.

In 1986, for the first time, the complete chloroplast genomes of tobacco (*Nicotiana tabacum* ^68^) and liverwort (*Marchantia polymorpha* ^69^) were obtained and the chloroplast genes were annotated by gene expression. With the expansion of the NCBI database, homology searches by Blastx and Blastn against the GenBank database have been used to annotate chloroplast genes^70^ for several years. Consequently, the gene names and data annotation information are inconsistent among different studies^15, 70^. In addition, it is possible that some hypothetical chloroplast open reading frames (ycfs) or open reading frames (ORFs), whose functions and features have been identified, were not updated in previous studies^70, 71^. DOGMA (Dual Organellar GenoMe Annotator) is a web-based annotation package that solves some of these problems, including typos, incorrect sequences and gene names in GenBank^70^. Therefore, protein-coding, ribosomal RNA (rRNA) and transfer RNA (tRNA) genes of all the collected chloroplast genomes were re-annotated using DOGMA with the default settings. However, because BLAST cannot provide a precise search for start and stop codons for the protein coding genes and those genes with more than one intron were annotated as two genes^70^, the start and stop codons must be chosen by manual operation. Thus, we further modified the annotation information using our own Perl scripts.

### Phylogenetic analysis

Chloroplast genomes were analyzed at the order and species level. We collected 45 orders, and the phylogenetic relationship of these orders was an integration of previously published phylogenies established by Jansen *et al*.^72^, Moore *et al*.^73^ and APG III^74^. For the species tree, maximum likelihood (ML) analyses were performed on datasets of 40 genes to ensure sufficient information for the calculation of branch length^75, 76^. An individual gene matrix was aligned using T-Coffee^77^ and then manually adjusted. We used group-to-group profile alignments^78, 79^ by taking advantage of previously recognized phylogenetic relationships^72–74^, which yielded data matrices with fewer missing data compared to other methods^79^. We then identified and concatenated alignment clusters of homologous gene regions. ML analysis was conducted using RAxML version 7.0.4^80^ using the PROTGAMMAJTT substitution model and default settings. Support for each node for ML analysis was tested with 1000 bootstrap replicates. These trees were viewed and edited with the TreeExploter program in MEGA 5.0^81^.

### Statistical tests based on cross-species and phylogenetic signal analysis

To identify the relationship between chloroplast genome size and all the other characteristics of chloroplast genome sequences shown in Supplementary Table S1, we conducted a conventional analysis of variance (ANOVA) to test the differences between genome size and all sequence characteristics based on cross-species and phylogenetic signal analyses^82^. In cross-species analysis, the relationship between each pair-wise characteristic and chloroplast genome size was described using their standardized major axes without taking phylogeny into account (SMA; model II regression). We computed the common slope using SMA analyses among species with a likelihood ratio procedure^83^. The smatr package^84^ of R^85^ was used to perform the SMA analyses.

The ANOVAs were carried out using the PDAP package to test whether there was significant cross-species association between sequence characteristics and genome size that could also be a small-probability event based on a random model of Brownian motion evolution^86^. We first used Pdsimul to generate 1000 Monte Carlo simulated data by taking the tree topology and branch length information into account (see the phylogenetic analysis section)^86^. The *F*-statistic of ANOVA of the simulated data was analyzed by pdanova, and the obtained values were compared against the observed *F*-statistic from the cross-species analysis. If the observed *F*-statistic was greater than 95% of the simulated data, the relationship between chloroplast genome size and other characteristics was not random and was affected by phylogenetic signals. This analysis was implemented separately for each characteristic. *K* was the descriptive statistical parameter to describe the degree of the difference between the *F*-statistic of simulated data and observed *F*-statistic distributions^87^. In brief, the *K* statistic was the ratio of the observed mean square error derived from a phylogenetically corrected mean and the expected mean square error obtained from the analysis by considering tree topology and branch length information based on a Brownian motion evolution model^82^. *K* = 1 would denote that the species had a close relationship with the same characteristic values as those obtained from a Brownian motion evolution model, whereas *K* < 1 would indicate that the relationship of the characteristic values was not affected by phylogenetic signals. Slope estimates and *r* ^*2*^ from SMA analyses^86^ were obtained from the results of our standardized contrasts utilizing pdtree and the R package smatr^85^. In addition, we performed the same likelihood ratio procedure as described earlier in this section to test the common slope for within-group SMA analyses^84^.

### Ratio of nonsynonymous to synonymous nucleotide substitutions (*Ka*/*Ks*)

The ratio of nonsynonymous to synonymous substitutions (*Ka*/*Ks*) of all individual datasets was estimated for each branch of the phylogenetic tree using PAML^88, 89^. A free-ratio model was implemented in PAML, and an independent *Ka*/*Ks* value was assumed separately for each branch. Because independent estimation of the *Ka*/*Ks* ratio for each branch of the tree was extremely time-consuming, the phylogenetic tree of angiosperms was divided into six monophyletic sub-trees, while the phylogenetic tree of gymnosperms was divided into three sub-trees, and each of the sub-trees was evaluated independently. A free-ratio model was implemented in PAML, and an independent *Ka*/*Ks* value was separately assumed for each branch^90, 91^. Only the *Ka*/*Ks* values between modern species and their most recent reconstructed ancestors were used in subsequent analyses. Thus, we focused only on the rate of accumulation of mutations between homologous gene pairs with the most recent common ancestors.

## Electronic supplementary material

Table S1 The basic information of chloroplast genome
