# Supplementary material for: Inferring the evolutionary mechanism of the chloroplast genome size by comparing whole-chloroplast genome sequences in seed plants
Source: Sci Rep. 2017 May 8;7:1555. doi: 10.1038/s41598-017-01518-5 (PMC5431534; doi:10.1038/s41598-017-01518-5)
Supplement: Supplementary file 1 — Table S1 The basic information of chloroplast genome [file 41598_2017_1518_MOESM1_ESM.pdf]

Inferring the evolutionary mechanism of the chloroplast genome size by comparing whole-chloroplast genome sequences in seed plants

Zheng Xiao-Ming[1,+], Wang Junrui[1,+], Feng Li[1], Liu Sha[1], Pang Hongbo[2], Qi Lan[1], Li Jing[1], Sun Yan[1], Qiao Weihua[1], Zhang Lifang[1], Cheng Yunlian[1], Yang Qingwen[1,*]

[1]National Key Facility for Crop Gene Resources and Genetic Improvement, Institute of Crop Sciences, Chinese Academy of Agricultural Sciences, Beijing 100081, China
[2]College of Chemistry and Life Science, Shenyang Normal University, Shenyang 110034, China
[*]yangqingwen@caas.cn
[+]These authors contributed equally to this work.

Table S1 The basic information of chloroplast genomes collected in this study.

Species Name	Family	Order	Order Abbrevia-tion	NC[1]	GC[2]	TL[3]	LSCL[4]	SSCL[5]	IRL[6]	GN[7]	IGRL[8]	GRL[9]
Asclepias nivea	Apocynaceae	Gentianales	Gent	NC_022431	37.92	161592	91871	18771	25475	130	78807	82785
Asclepias syriaca	Apocynaceae	Gentianales	Gent	NC_022432	37.83	158719	89347	18572	25400	128	74836	83883
Coffea arabica	Rubiaceae	Gentianales	Gent	NC_008535	37.00	155189	85166	18137	25943	130	61484	93705
Catharanthus roseus	Apocynaceae	Gentianales	Gent	NC_021423	37.83	154950	85765	17997	25594	133	62268	92682
Sesamum indicum	Pedaliaceae	Lamiales	Lami	NC_016433	38.20	153324	85170	17872	25141	132	61893	91431
Boea hygrometrica	Gesneriaceae	Lamiales	Lami	NC_016468	37.59	153493	84698	17903	25446	129	61370	92123
Jasminum nudiflorum	Oleaceae	Lamiales	Lami	NC_008407	38.00	165121	92877	13272	29486	133	93435	71686
Olea woodiana subsp. woodiana	Oleaceae	Lamiales	Lami	NC_015608	37.79	155942	86711	17799	25716	130	63287	92655
Olea europaea subsp. cuspidata	Oleaceae	Lamiales	Lami	NC_015604	37.81	155862	86612	17788	25731	130	63231	92631
Olea europaea subsp. maroccana	Oleaceae	Lamiales	Lami	NC_015623	37.81	155896	86623	17789	25742	130	63238	92658
Cistanche deserticola	Orobanchaceae	Lamiales	Lami	NC_021111	36.80	102657	49130	8819	22354	109	54272	48385
Tectona grandis	Lamiaceae	Lamiales	Lami	NC_020098	37.89	153953	85318	17741	25447	131	61210	92743
Olea europaea	Oleaceae	Lamiales	Lami	NC_013707	37.80	155888	86590	17814	25742	130	63215	92673
Utricularia gibba	Lentibulariaceae	Lamiales	Lami	NC_021449	37.57	152113	81818	14487	27904	132	62519	89594
Salvia miltiorrhiza	Lamiaceae	Lamiales	Lami	NC_020431	38.00	151328	82695	17555	25539	131	58224	93104
Epifagus virginiana	Orobanchaceae	Lamiales	Lami	NC_001568	36.00	70028	19799	4759	22735	56	27551	42477

Solanum bulbocastanum	Solanaceae	Solanales	Sola	NC_007943	37.88	155371	85814	18381	25588	138	62474	92897
Solanum tuberosum	Solanaceae	Solanales	Sola	NC_008096	37.90	155312	85749	18373	25595	137	62424	92888
Solanum lycopersicum	Solanaceae	Solanales	Sola	NC_007898	37.86	155461	85882	18363	25611	140	62555	92906
Datura stramonium	Solanaceae	Solanales	Sola	NC_018117	37.88	155871	86299	18368	25602	133	62932	92939
Capsicum annuum	Solanaceae	Solanales	Sola	NC_018552	37.70	156781	87366	25783	17849	132	63606	93175
Atropa belladonna	Solanaceae	Solanales	Sola	NC_004561	37.60	156688	86868	18008	25906	130	63436	93252
Cuscuta exaltata	Convolvulaceae	Solanales	Sola	NC_009963	38.12	125373	82721	9250	16701	110	55537	69836
Nicotiana undulata	Solanaceae	Solanales	Sola	NC_016068	37.88	155863	86633	18568	25331	155	63067	92796
Nicotiana sylvestris	Solanaceae	Solanales	Sola	NC_007500	37.90	155941	86684	18573	25342	146	63164	92777
Nicotiana tomentosiformis	Solanaceae	Solanales	Sola	NC_007602	37.80	155745	86392	18495	25429	147	62880	92865
Cuscuta reflexa	Convolvulaceae	Solanales	Sola	NC_009766	38.22	121521	79468	8571	16741	112	53505	68016
Ipomoea purpurea	Convolvulaceae	Solanales	Sola	NC_009808	37.48	162046	88172	12110	30882	131	75869	86177
Panax ginseng	Araliaceae	Apiales	Apia	NC_006290	38.00	156318	86106	18070	26071	131	63316	93002
Eleutherococcus senticosus	Araliaceae	Apiales	Apia	NC_016430	38.00	156768	86755	18153	25930	131	63988	92780
Daucus carota	Apiaceae	Apiales	Apia	NC_008325	37.66	155911	84242	17567	27051	136	64023	91888
Anthriscus cerefolium	Apiaceae	Apiales	Apia	NC_015113	37.42	154719	84774	17551	26197	130	62048	92671
Lactuca sativa	Asteraceae	Asterales	Aste	NC_007578	38.00	152772	84105	18599	25034	129	66161	86611
Ageratina adenophora	Asteraceae	Asterales	Aste	NC_015621	37.50	150689	84815	18358	23755	137	61890	88799

Trachelium caeruleum	Campanulaceae	Asterales	Aste	NC_010442	38.30	162321	100114	7661	27273	132	90675	71646
Jacobaea vulgaris	Asteraceae	Asterales	Aste	NC_015543	37.32	150686	82855	18277	24777	132	64319	86367
Artemisia frigida	Asteraceae	Asterales	Aste	NC_020607	37.48	151076	82740	18394	24971	132	64332	86744
Chrysanthemum x morifolium	Asteraceae	Asterales	Aste	NC_020092	37.48	151033	82782	18345	24953	128	63506	87527
Chrysanthemum indicum	Asteraceae	Asterales	Aste	NC_020320	37.48	150972	82731	18327	24957	125	63516	87456
Helianthus annuus	Asteraceae	Asterales	Aste	NC_007977	38.00	151104	83530	18308	24633	131	65195	85909
Guizotia abyssinica	Asteraceae	Asterales	Aste	NC_010601	37.62	151762	83527	18237	24999	130	65155	86607
Parthenium argentatum	Asteraceae	Asterales	Aste	NC_013553	37.60	152803	84335	19390	24424	142	65021	87782
Camellia sinensis	Theaceae	Ericales	Eric	NC_020019	37.31	157103	86645	18276	26091	132	63588	93515
Camellia taliensis	Theaceae	Ericales	Eric	NC_022264	37.32	156974	86672	18240	26031	137	63548	93426
Ardisia polysticta	Primulaceae	Ericales	Eric	NC_021121	37.07	156506	86078	18328	26050	132	63634	92872
Silene vulgaris	Caryophyllaceae	Caryophyllales	Cary	NC_016727	36.25	151583	82258	17309	26008	126	58053	93530
Silene latifolia	Caryophyllaceae	Caryophyllales	Cary	NC_016730	36.43	151736	82704	17220	25906	126	58248	93488
Silene noctiflora	Caryophyllaceae	Caryophyllales	Cary	NC_016728	36.51	151639	79475	12382	29891	127	68729	82910
Silene conica	Caryophyllaceae	Caryophyllales	Cary	NC_016729	36.12	147208	80129	13363	26858	126	66671	80537
Fagopyrum esculentum	Polygonaceae	Caryophyllales	Cary	NC_010776	38.00	159599	84888	13343	30684	129	75254	84345
Barbarea verna	Brassicaceae	Brassicales	Bras	NC_009269	36.43	154532	83435	18093	26502	130	67838	86694
Nasturtium officinale	Brassicaceae	Brassicales	Bras	NC_009275	36.37	155105	84275	17832	26499	130	67686	87419
Arabidopsis thaliana	Brassicaceae	Brassicales	Bras	NC_000932	36.30	154478	84170	17780	26264	129	68117	86361

Lobularia maritima	Brassicaceae	Brassicales	Bras	NC_009274	36.51	152659	82399	17668	26296	129	66265	86394
Arabis hirsuta	Brassicaceae	Brassicales	Bras	NC_009268	36.40	153689	82819	18036	26417	129	66272	87417
Draba nemorosa	Brassicaceae	Brassicales	Bras	NC_009272	36.47	153289	82473	18126	26345	129	66224	87065
Aethionemagrandiflorum	Brassicaceae	Brassicales	Bras	NC_009266	36.81	154243	83488	17765	26495	129	66841	87402
Aethionema cordifolium	Brassicaceae	Brassicales	Bras	NC_009265	36.62	154168	83418	17742	26504	129	67664	86504
Carica papaya	Caricaceae	Brassicales	Bras	NC_010323	36.89	160100	88749	18701	26325	129	67059	93041
Brassica napus	Brassicaceae	Brassicales	Bras	NC_016734	36.30	152850	83030	17760	26035	131	66557	86293
Olimarabidopsis pumila	Brassicaceae	Brassicales	Bras	NC_009267	36.48	154737	83883	17878	26488	130	67804	86933
Capsella bursa-pastoris	Brassicaceae	Brassicales	Bras	NC_009270	36.55	154490	83741	17847	26451	130	66967	87523
Crucihimalaya wallichii	Brassicaceae	Brassicales	Bras	NC_009271	36.42	155199	84097	18064	26519	130	68947	86252
Lepidium virginicum	Brassicaceae	Brassicales	Bras	NC_009273	36.47	154743	84035	17988	26360	130	68187	86556
Pachycladon enysii	Brassicaceae	Brassicales	Bras	NC_018565	36.39	154896	83925	18067	26452	129	67769	87127
Pachycladon cheesemanii	Brassicaceae	Brassicales	Bras	NC_021102	36.43	154498	83550	17968	26490	130	67412	87086
Gossypium darwinii	Malvaceae	Malvales	Malv	NC_016670	37.20	160378	88906	20266	25603	130	68292	92086
Gossypium herbaceum	Malvaceae	Malvales	Malv	NC_016692	37.20	160315	88790	20285	25620	130	68531	91784
Gossypium thurberi	Malvaceae	Malvales	Malv	NC_015204	37.22	160264	88737	20271	25628	133	68175	92089
Gossypium hirsutum	Malvaceae	Malvales	Malv	NC_007944	37.25	160301	88816	20269	25608	130	68203	92098

Gossypium tomentosum	Malvaceae	Malvales	Malv	NC_016690	37.20	160433	88932	20271	25615	130	68335	92098
Gossypium mustelinum	Malvaceae	Malvales	Malv	NC_016711	37.20	160313	88826	20269	25609	130	68208	92105
Gossypium barbadense	Malvaceae	Malvales	Malv	NC_008641	37.23	160317	88841	20294	25591	123	68394	91923
Gossypium arboreum	Malvaceae	Malvales	Malv	NC_016712	37.20	160230	88722	20274	25617	130	68140	92090
Gossypium raimondii	Malvaceae	Malvales	Malv	NC_016668	37.20	160161	88656	20205	25650	130	68457	91704
Gossypium gossypioides	Malvaceae	Malvales	Malv	NC_017894	37.20	159959	88803	20004	25576	130	68317	91642
Gossypium capitis-viridis	Malvaceae	Malvales	Malv	NC_018111	37.32	159467	88065	20198	25602	128	67836	91631
Gossypium incanum	Malvaceae	Malvales	Malv	NC_018109	37.39	159205	87879	20196	25565	128	67663	91542
Gossypium somalense	Malvaceae	Malvales	Malv	NC_018110	37.37	159539	88150	20251	25569	128	67913	91626
Gossypium areysianum	Malvaceae	Malvales	Malv	NC_018112	37.37	159572	88182	20252	25569	128	67946	91626
Gossypium robinsonii	Malvaceae	Malvales	Malv	NC_018113	37.16	159849	88486	20203	25580	128	68271	91578
Theobroma cacao	Malvaceae	Malvales	Malv	NC_014676	36.90	160604	89395	20187	25511	126	68393	92211
Citrus sinensis	Rutaceae	Sapindales	Sapi	NC_008334	38.48	160129	87744	18393	26996	140	72536	87593
Eucalyptus obliqua	Myrtaceae	Myrtales	Myrt	NC_022378	36.90	159527	88293	18498	26368	130	66255	93272
Eucalyptus radiata	Myrtaceae	Myrtales	Myrt	NC_022379	36.90	159529	88295	18498	26368	130	66194	93335
Eucalyptus delegatensis	Myrtaceae	Myrtales	Myrt	NC_022380	36.87	159724	88448	18540	26368	130	66368	93356

Eucalyptus verrucata	Myrtaceae	Myrtales	Myrt	NC_022381	36.80	160109	88890	18481	26369	130	66823	93286
Eucalyptus umbra	Myrtaceae	Myrtales	Myrt	NC_022387	36.84	159576	88864	18658	26027	130	66591	92985
Eucalyptus patens	Myrtaceae	Myrtales	Myrt	NC_022389	36.82	160187	88902	18543	26371	130	66856	93331
Eucalyptus baxteri	Myrtaceae	Myrtales	Myrt	NC_022382	36.82	160032	88926	18368	26369	130	66667	93365
Eucalyptus diversifolia	Myrtaceae	Myrtales	Myrt	NC_022383	36.83	159954	88901	18315	26369	130	66649	93305
Eucalyptus cloeziana	Myrtaceae	Myrtales	Myrt	NC_022388	36.85	160015	88867	18446	26351	130	66746	93269
Eucalyptus marginata	Myrtaceae	Myrtales	Myrt	NC_022390	36.84	160076	88828	18476	26386	130	66742	93334
Eucalyptus regnans	Myrtaceae	Myrtales	Myrt	NC_022386	36.82	160031	88860	18447	26362	130	66740	93291
Eucalyptus sieberi	Myrtaceae	Myrtales	Myrt	NC_022384	36.83	159985	88848	18401	26368	130	66628	93357
Eucalyptus elata	Myrtaceae	Myrtales	Myrt	NC_022385	36.85	159899	88762	18401	26368	130	66566	93333
Eucalyptus salmonophloia	Myrtaceae	Myrtales	Myrt	NC_022403	36.83	160413	89173	18466	26387	130	67094	93319
Eucalyptus microcorys	Myrtaceae	Myrtales	Myrt	NC_022404	36.83	160225	89051	18410	26382	130	66900	93325
Eucalyptus guilfoylei	Myrtaceae	Myrtales	Myrt	NC_022405	36.86	160520	89054	18096	26685	130	66960	93560
Eucalyptus polybractea	Myrtaceae	Myrtales	Myrt	NC_022393	36.85	160268	88944	18530	26397	130	67007	93261
Eucalyptus cladocalyx	Myrtaceae	Myrtales	Myrt	NC_022394	36.86	160213	89045	18376	26396	130	66892	93321
Eucalyptus deglupta	Myrtaceae	Myrtales	Myrt	NC_022399	36.87	160177	88936	18425	26408	130	66817	93360
Eucalyptus melliodora	Myrtaceae	Myrtales	Myrt	NC_022392	36.84	160386	89073	18557	26378	130	67158	93228
Eucalyptus torquata	Myrtaceae	Myrtales	Myrt	NC_022401	36.86	160223	89018	18439	26383	130	67022	93201
Eucalyptus diversicolor	Myrtaceae	Myrtales	Myrt	NC_022402	36.84	160214	88994	18416	26402	130	66942	93272
Eucalyptus spathulata	Myrtaceae	Myrtales	Myrt	NC_022400	36.83	161071	88729	17116	27613	130	66480	94591
Eucalyptus grandis	Myrtaceae	Myrtales	Myrt	NC_014570	36.89	160137	88872	18475	26395	118	69945	90192
Eucalyptus camaldulensis	Myrtaceae	Myrtales	Myrt	NC_022398	36.87	160164	88874	18492	26399	130	66803	93361
Eucalyptus globulus	Myrtaceae	Myrtales	Myrt	NC_008115	36.85	160286	89012	18488	26393	130	66985	93301

Eucalyptus saligna	Myrtaceae	Myrtales	Myrt	NC_022397	36.85	160015	89041	18426	26274	130	66939	93076
Eucalyptus nitens	Myrtaceae	Myrtales	Myrt	NC_022395	36.86	160271	89005	18468	26399	130	66936	93335
Eucalyptus aromaphloia	Myrtaceae	Myrtales	Myrt	NC_022396	36.86	160149	88925	18468	26378	130	66852	93297
Eucalyptus curtisii	Myrtaceae	Myrtales	Myrt	NC_022391	36.98	160038	88828	18448	26381	130	66702	93336
Eucalyptus erythrocorys	Myrtaceae	Myrtales	Myrt	NC_022406	36.87	159742	88691	18287	26382	130	66397	93345
Allosyncarpia ternata	Myrtaceae	Myrtales	Myrt	NC_022413	36.82	159593	88218	18571	26402	130	66383	93210
Stockwellia quadrifida	Myrtaceae	Myrtales	Myrt	NC_022414	36.85	159561	88247	18544	26385	130	66532	93029
Corymbia gummifera	Myrtaceae	Myrtales	Myrt	NC_022407	36.75	160713	88310	17197	27603	130	66236	94477
Corymbia eximia	Myrtaceae	Myrtales	Myrt	NC_022409	36.79	160012	88522	18672	26409	130	66760	93252
Corymbia maculata	Myrtaceae	Myrtales	Myrt	NC_022408	36.79	160045	88557	18670	26409	130	66793	93252
Corymbia tessellaris	Myrtaceae	Myrtales	Myrt	NC_022410	36.77	160127	88617	18692	26409	130	66872	93255
Angophora floribunda	Myrtaceae	Myrtales	Myrt	NC_022411	36.76	160245	88715	18746	26392	130	67057	93188
Angophora costata	Myrtaceae	Myrtales	Myrt	NC_022412	36.76	160326	88769	18773	26392	130	67138	93188
Oenothera argillicola	Onagraceae	Myrtales	Myrt	NC_010358	39.13	165055	88511	19000	28772	130	71580	93475
Oenothera glazioviana	Onagraceae	Myrtales	Myrt	NC_010360	39.01	165225	89591	18882	28376	130	71844	93381
Oenothera biennis	Onagraceae	Myrtales	Myrt	NC_010361	39.07	164807	88964	18901	28471	130	71525	93282
Oenothera parviflora	Onagraceae	Myrtales	Myrt	NC_010362	39.06	163365	87732	18895	28369	130	70247	93118
Pelargonium x hortorum	Geraniaceae	Geraniales	Gera	NC_008454	39.60	217942	59710	6750	75741	161	91847	126095

Erodium carvifolium	Geraniaceae	Geraniales	Gera	NC_015083	39.00	116934	/[10]	/	/	108	54041	62893
Glycine cyrtoloba	Fabaceae	Fabales	Faba	NC_021647	35.33	152518	83579	17879	25530	127	61813	90705
Glycine dolichocarpa	Fabaceae	Fabales	Faba	NC_021648	35.31	152804	83819	17823	25581	127	62060	90744
Glycine syndetika	Fabaceae	Fabales	Faba	NC_021650	35.32	152783	83839	17874	25535	127	62075	90708
Glycine tomentella	Fabaceae	Fabales	Faba	NC_021636	35.33	152728	83777	17865	25543	127	62023	90705
Glycine stenophita	Fabaceae	Fabales	Faba	NC_021649	35.33	153023	84033	17884	25553	127	62333	90690
Glycine cyrtoloba	Fabaceae	Fabales	Faba	NC_021645	35.31	152381	83574	17837	25485	126	63857	88524
Glycine stenophita	Fabaceae	Fabales	Faba	NC_021646	35.32	152618	83941	24377	22150	127	61969	90649
Glycine max	Fabaceae	Fabales	Faba	NC_007942	35.37	152218	83175	17895	25574	128	61757	90461
Phaseolus vulgaris	Fabaceae	Fabales	Faba	NC_009259	35.44	150285	79824	17610	26426	127	63859	86426
Vigna unguiculata	Fabaceae	Fabales	Faba	NC_018051	35.24	152415	80764	18483	26584	130	64833	87582
Vigna radiata	Fabaceae	Fabales	Faba	NC_013843	35.18	151271	80896	17427	26474	127	64858	86413
Vigna angularis	Fabaceae	Fabales	Faba	NC_021091	35.19	151683	81028	17464	26460	127	65075	86608
Millettia pinnata	Fabaceae	Fabales	Faba	NC_016708	34.80	152968	83401	18511	25528	131	61638	91330
Lotus japonicus	Fabaceae	Fabales	Faba	NC_002694	36.00	150519	81936	18271	25156	127	58572	91947
Cicer arietinum	Fabaceae	Fabales	Faba	NC_011163	33.90	125319	/	/	/	108	57781	67538
Medicago truncatula	Fabaceae	Fabales	Faba	NC_003119	33.97	124033	/	/	/	109	57782	66251
Trifolium subterraneum	Fabaceae	Fabales	Faba	NC_011828	34.00	144763	/	/	/	111	78324	66439
Pisum sativum	Fabaceae	Fabales	Faba	NC_014057	34.83	122169	/	/	/	108	56545	65624
Lathyrus sativus	Fabaceae	Fabales	Faba	NC_014063	35.11	121020	/	/	/	108	55868	65152
Fragaria chiloensis	Rosaceae	Rosales	Rosa	NC_019601	37.22	155603	85567	18146	25945	130	62683	92920
Fragaria virginiana	Rosaceae	Rosales	Rosa	NC_019602	37.23	155621	85586	18145	25945	130	62701	92920

Fragaria vesca subsp. vesca	Rosaceae	Rosales	Rosa	NC_015206	37.21	155691	85606	18173	25956	130	62762	92929
Fragaria vesca subsp. bracteata	Rosaceae	Rosales	Rosa	NC_018766	36.13	129788	/	/	/	111	53664	76124
Prunus persica	Rosaceae	Rosales	Rosa	NC_014697	36.80	157790	85968	19060	26381	130	65233	92557
Pyrus pyrifolia	Rosaceae	Rosales	Rosa	NC_015996	36.58	159922	87901	19237	26392	130	67343	92579
Morus indica	Moraceae	Rosales	Rosa	NC_008359	36.37	158484	87386	19742	25678	129	66144	92340
Pentactina rupicola	Rosaceae	Rosales	Rosa	NC_016921	36.80	156612	84970	18940	26351	129	63437	93175
Castanea mollissima	Fagaceae	Fagales	Faga	NC_014674	36.80	160799	90432	18995	25686	128	68173	92626
Cucumis sativus	Cucurbitaceae	Cucurbitales	Cucu	NC_007144	37.08	155293	86688	18222	25191	130	69375	85918
Corynocarpus laevigata	Corynocarpaceae	Cucurbitales	Cucu	NC_014807	36.56	159202	88862	18992	25674	127	66713	92489
Cucumis melo	Cucurbitaceae	Cucurbitales	Cucu	NC_015983	36.90	156017	86334	18090	25797	132	68662	87355
Manihot esculenta	Euphorbiaceae	Malphigiales	Malp	NC_010433	35.87	161453	89295	18250	26954	129	73677	87776
Hevea brasiliensis	Euphorbiaceae	Malphigiales	Malp	NC_015308	35.74	161191	89209	18362	26810	128	67976	93215
Ricinus communis	Euphorbiaceae	Malphigiales	Malp	NC_016736	35.74	163161	89652	18817	27347	131	75223	87938
Jatropha curcas	Euphorbiaceae	Malphigiales	Malp	NC_012224	35.36	163856	91756	17852	27124	130	70289	93567
Populus alba	Salicaceae	Malpighiales	Malp	NC_008235	36.74	156505	84618	16567	27660	128	63245	93260
Populus trichocarpa	Salicaceae	Malpighiales	Malp	NC_009143	36.68	157033	85129	16600	27652	143	63789	93244
Vitis vinifera	Vitaceae	Vitaceae	Vita	NC_007957	37.40	160928	89147	19065	26358	137	67832	93096
Buxus microphylla	Buxaceae	Buxales	Buxa	NC_009599	38.10	159010	88143	17747	26560	139	65825	93185
Tetracentron sinense	Trochodendraceae	Trochodendrales	Troc	NC_021425	38.10	164467	84466	19539	30231	137	68530	95937
Trochodendron aralioides	Trochodendraceae	Trochodendrales	Troc	NC_021426	38.00	165945	85483	18974	30744	137	69408	96537

Nelumbo lutea	Nelumbonaceae	Proteales	Prot	NC_015605	38.01	163206	91760	19340	26053	130	69726	93480
Nelumbo nucifera	Nelumbonaceae	Proteales	Prot	NC_015610	37.99	163307	91847	19330	26065	130	69728	93579
Platanus occidentalis	Platanaceae	Proteales	Prot	NC_008335	38.00	161791	92150	19509	25066	131	69251	92540
Ranunculus macranthus	Ranunculaceae	Ranunculales	Ranu	NC_008796	37.90	155129	84638	18909	25791	129	68870	86259
Megaleranthis saniculifolia	Ranunculaceae	Ranunculales	Ranu	NC_012615	38.00	159924	88326	18382	26608	132	66920	93004
Nandina domestica	Berberidaceae	Ranunculales	Ranu	NC_008336	38.30	156599	85473	19002	26062	131	63470	93129
Phyllostachys nigra	Poaceae	Poales	Poal	NC_015826	38.90	139839	83234	12879	21863	131	62904	76935
Phyllostachys propinqua	Poaceae	Poales	Poal	NC_016699	38.88	139704	83227	12877	21800	129	62919	76785
Phyllostachys edulis	Poaceae	Poales	Poal	NC_015817	38.90	139679	83213	12870	21798	131	62874	76805
Acidosasa purpurea	Poaceae	Poales	Poal	NC_015820	38.90	139697	83273	12834	21795	131	62812	76885
Arundinaria gigantea	Poaceae	Poales	Poal	NC_020341	38.93	138935	82632	12709	21797	131	61816	77119
Dendrocalamus latiflorus	Poaceae	Poales	Poal	NC_013088	38.90	139365	83001	12854	21755	132	63424	75941
Bambusa emeiensis	Poaceae	Poales	Poal	NC_015830	38.90	139493	82988	12901	21802	131	62375	77118
Brachypodium distachyon	Poaceae	Poales	Poal	NC_011032	38.57	135197	79446	12668	21540	127	60095	75102
Triticum aestivum	Poaceae	Poales	Poal	NC_002762	38.31	134545	80349	12790	20703	133	59374	75171
Hordeum vulgare	Poaceae	Poales	Poal	NC_008590	38.31	136462	80600	12704	21579	139	60099	76363
Agrostis stolonifera	Poaceae	Poales	Poal	NC_008591	38.45	136584	80546	12740	21649	133	59634	76950
Lolium perenne	Poaceae	Poales	Poal	NC_009950	38.25	135282	79964	12428	21445	130	59966	75316
Festuca arundinacea	Poaceae	Poales	Poal	NC_011713	38.39	136048	80560	11300	22600	126	61779	74269
Oryza nivara	Poaceae	Poales	Poal	NC_005973	39.00	134494	80544	12346	20802	165	59123	75371

Oryza sativa	Poaceae	Poales	Poal	NC_008155	39.00	134496	80553	12347	20798	159	59130	75366
Panicum virgatum	Poaceae	Poales	Poal	NC_015990	38.59	139677	81729	12540	22704	133	64163	75514
Zea mays	Poaceae	Poales	Poal	NC_001666	38.50	140387	82355	12536	22748	157	62980	77407
Triticum monococcum	Poaceae	Poales	Poal	NC_021760	37.37	116399	/	/	/	113	51631	64768
Triticum urartu	Poaceae	Poales	Poal	NC_021762	37.38	115773	/	/	/	92	51409	64364
Aegilops tauschii	Poaceae	Poales	Poal	NC_022133	37.26	114112	/	/	/	114	49418	64694
Aegilops speltoides	Poaceae	Poales	Poal	NC_022135	37.26	113536	/	/	/	111	49465	64071
Saccharum hybrid cultivar	Poaceae	Poales	Poal	NC_006084	38.40	141182	83048	12544	22795	163	64488	76694
Coix lacryma-jobi	Poaceae	Poales	Poal	NC_013273	38.46	145745	82792	12523	22715	152	64210	81535
Sorghum bicolor	Poaceae	Poales	Poal	NC_008602	38.50	140754	82688	12502	22782	140	64078	76676
Pharus latifolius	Poaceae	Poales	Poal	NC_021372	38.37	142077	83340	12531	23103	117	63991	78086
Typha latifolia	Typhaceae	Poales	Poal	NC_013823	33.80	161572	89140	19652	26390	131	68192	93380
Anomochloa marantoidea	Poaceae	Poales	Poal	NC_014062	38.66	138412	82274	12162	21988	130	64150	74262
Oryza meridionalis	Poaceae	Poales	Poal	NC_016927	39.01	134558	80605	12347	20803	124	59190	75368
Oryza rufipogon	Poaceae	Poales	Poal	NC_017835	39.00	134544	80594	12348	20801	122	59174	75370
Elaeis guineensis	Arecaceae	Arecales	Arec	NC_017602	37.40	156973	85192	17639	27071	132	63316	93657
Cocos nucifera	Arecaceae	Arecales	Arec	NC_022417	37.44	154731	84230	17391	26555	131	61263	93468
Phoenix dactylifera	Arecaceae	Arecales	Arec	NC_013991	37.23	158462	86198	17712	27276	147	64459	94003
Cymbidium aloifolium	Orchidaceae	Asparagales	Aspa	NC_021429	36.87	156904	85641	17871	26696	124	65336	91568
Cymbidium mannii	Orchidaceae	Asparagales	Aspa	NC_021433	36.94	155308	85212	16666	26715	124	64848	90460

Cymbidium tracyanum	Orchidaceae	Asparagales	Aspa	NC_021432	36.80	156286	84968	17920	26699	124	63486	92800
Cymbidium sinense	Orchidaceae	Asparagales	Aspa	NC_021430	37.03	155548	84920	17670	26479	124	64698	90850
Cymbidium tortisepalum	Orchidaceae	Asparagales	Aspa	NC_021431	37.03	155627	84976	17709	26471	124	64786	90841
Oncidium hybrid cultivar	Orchidaceae	Asparagales	Aspa	NC_014056	37.32	146484	82324	12650	25755	119	63319	83165
Erycina pusilla	Orchidaceae	Asparagales	Aspa	NC_018114	36.65	143164	84189	12097	23439	126	65751	77413
Phalaenopsis aphrodite	Orchidaceae	Asparagales	Aspa	NC_007499	35.60	148964	85957	11543	25732	129	63954	85010
Phalaenopsis equestris	Orchidaceae	Asparagales	Aspa	NC_017609	36.65	148959	85967	11300	25846	119	67412	81547
Dioscorea elephantipes	Dioscoreaceae	Dioscoreales	Dios	NC_009601	38.90	152609	82777	18806	25513	129	60581	92028
Wolffiella lingulata	Araceae	Alismatales	Alis	NC_015894	35.84	169337	92015	13956	31683	128	71536	97801
Wolffia australiana	Araceae	Alismatales	Alis	NC_015899	35.86	168704	91454	13390	31930	128	70693	98011
Lemna minor	Araceae	Alismatales	Alis	NC_010109	35.70	165955	89906	13603	31223	130	67917	98038
Spirodela polyrhiza	Araceae	Alismatales	Alis	NC_015891	35.69	168788	91222	14056	31755	128	70770	98018
Colocasia esculenta	Araceae	Alismatales	Alis	NC_016753	36.20	162424	89670	22208	25273	131	70126	92298
Elodea canadensis	Hydrocharitaceae	Alismatales	Alis	NC_018541	37.00	156700	86194	17810	26348	129	70994	85706
Najas flexilis	Hydrocharitaceae	Alismatales	Alis	NC_021936	38.20	156329	88668	5266	31198	119	87919	68410
Acorus americanus	Acoraceae	Acorales	Acor	NC_010093	38.59	153819	83496	18273	26025	130	62550	91269
Calycanthus floridus	Calycanthaceae	Laurales	Laur	NC_004993	39.27	153337	86948	19797	23296	132	61844	91493

Magnolia officinalis	Magnoliaceae	Magnoliales	Magn	NC_020316	39.22	160183	88210	18843	26565	127	66774	93409
Magnolia officinalis	Magnoliaceae	Magnoliales	Magn	NC_020317	39.24	160105	88186	18843	26538	129	66620	93485
Magnolia grandiflora	Magnoliaceae	Magnoliales	Magn	NC_020318	39.30	159623	87757	18740	26563	129	66239	93384
Magnolia kwangsiensis	Magnoliaceae	Magnoliales	Magn	NC_015892	39.26	159667	88030	18669	26484	129	66324	93343
Magnolia denudata	Magnoliaceae	Magnoliales	Magn	NC_018357	39.25	160053	88101	18772	26590	129	66603	93450
Liriodendron tulipifera	Magnoliaceae	Magnoliales	Magn	NC_008326	39.16	159886	88150	18964	26386	129	66814	93072
Drimys granadensis	Winteraceae	Canellales	Cane	NC_008456	38.79	160604	88685	18621	26649	137	66816	93788
Piper cenocladum	Piperaceae	Piperales	Pipe	NC_008457	38.31	160624	87668	18878	27039	130	70859	89765
Chloranthus spicatus	Chloranthaceae	Chloranthales	Chlo	NC_009598	37.10	157772	88108	18398	26133	133	64287	93485
Illicium oligandrum	Schisandraceae	Austrobaileyales	Aust	NC_009600	39.00	148552	98057	20267	15114	126	64570	83982
Nymphaea alba	Nymphaeaceae	Nymphaeales	Nymp	NC_006050	39.15	159930	90014	19562	25177	130	74040	85890
Nuphar advena	Nymphaeaceae	Nymphaeales	Nymp	NC_008788	39.10	160866	90379	18817	25835	131	74834	86032
Trithuria inconspicua	Hydatellaceae	Nymphaeales	Nymp	NC_020372	39.56	165389	84468	6367	37277	139	88706	76683
Amborella trichopoda	Amborellaceae	Amborellales	Ambo	NC_005086	38.34	162686	90970	18414	26651	128	74802	87884
Cycas taitungensis	Cycadaceae	Cycadales	Cyca	NC_009618	39.50	163403	90216	23039	25074	133	87902	75501
Cycas revoluta	Cycadaceae	Cycadales	Cyca	NC_020319	39.38	162489	88978	23379	25066	156	86991	75498
Ginkgo biloba	Ginkgoaceae	Ginkgoales	Gink	NC_016986	39.56	156982	99251	22267	17732	131	81822	75160
Gnetum parvifolium	Gnetaceae	Gnetales	Gnet	NC_011942	38.20	114914	66076	9538	19650	114	74391	40523
Gnetum montanum	Gnetaceae	Gnetales	Gnet	NC_021438	38.16	115019	66697	9494	19414	105	73744	41275
Welwitschia mirabilis	Welwitschiaceae	Welwitschiales	Welw	NC_010654	36.80	118919	68616	11059	19622	118	75812	43107
Ephedra equisetina	Ephedraceae	Ephedrales	Ephe	NC_011954	36.60	109518	59906	8104	20754	117	64107	45411

Pinus koraiensis	Pinaceae	Pinales	Pina	NC_004677	38.80	117190	/	/	/	113	53620	63570
Pinus lambertiana	Pinaceae	Pinales	Pina	NC_011156	38.69	117239	/	/	/	108	49018	68221
Pinus gerardiana	Pinaceae	Pinales	Pina	NC_011154	38.68	117618	/	/	/	108	54167	63451
Pinus krempfii	Pinaceae	Pinales	Pina	NC_011155	38.74	116989	/	/	/	105	50031	66958
Pinus monophylla	Pinaceae	Pinales	Pina	NC_011158	38.58	116479	/	/	/	108	54322	62157
Pinus nelsonii	Pinaceae	Pinales	Pina	NC_011159	38.61	116834	/	/	/	108	53845	62989
Pinus taeda	Pinaceae	Pinales	Pina	NC_021440	38.50	121530	/	/	/	109	51411	70119
Pinus thunbergii	Pinaceae	Pinales	Pina	NC_001631	38.50	119707	/	/	/	115	50525	69182
Pinus massoniana	Pinaceae	Pinales	Pina	NC_021439	38.55	119739	/	/	/	109	50387	69352
Picea sitchensis	Pinaceae	Pinales	Pina	NC_011152	37.59	120176	/	/	/	96	56104	64072
Picea morrisonicola	Pinaceae	Pinales	Pina	NC_016069	38.79	124168	/	/	/	112	57963	66205
Picea abies	Pinaceae	Pinales	Pina	NC_021456	37.90	124084	/	/	/	114	59997	64087
Keteleeria davidiana	Pinaceae	Pinales	Pina	NC_011930	38.60	117720	/	/	/	113	49196	68524
Cedrus deodara	Pinaceae	Pinales	Pina	NC_014575	39.10	119299	/	/	/	114	50519	68780
Larix decidua	Pinaceae	Pinales	Pina	NC_016058	38.78	122474	/	/	/	110	53330	69144
Pseudotsuga sinensis	Pinaceae	Pinales	Pina	NC_016064	38.76	122513	/	/	/	113	52293	70220
Podocarpus totara	Podocarpaceae	Podocarpales	Podo	NC_020361	37.16	133259	/	/	/	94	68231	65028
Cephalotaxus wilsoniana	Cephalotaxaceae	Cephalotaxales	Ceph	NC_016063	35.08	136196	/	/	/	113	72265	63931
Cephalotaxus oliveri	Cephalotaxaceae	Cephalotaxales	Ceph	NC_021110	35.24	134337	/	/	/	113	70416	63921
Cunninghamia lanceolata	Cupressaceae	Pinales	Pina	NC_021437	35.00	135334	/	/	/	116	71898	63436
Cryptomeria japonica	Cupressaceae	Pinales	Pina	NC_010548	35.40	131810	/	/	/	116	67816	63994

Taiwania cryptomerioides	Cupressaceae	Pinales	Pina	NC_016065	34.60	132588	/	/	/	121	70513	62075
Taiwania flousiana	Cupressaceae	Pinales	Pina	NC_021441	34.72	131413	/	/	/	107	69958	61455
Cathaya argyrophylla	Pinaceae	Pinales	Pina	NC_014589	38.80	107122	/	/	/	106	46110	61012
Taxus mairei	Taxaceae	Taxales	Taxa	NC_020321	34.72	127665	/	/	/	112	64452	63213

1: NC indicates Number from NCBI.
2: GC indicates GC content of a chloroplast genome.
3: TL indicates total length of chloroplast genome.
4: LSCL indicates length of large single copy.
5: SSCL indicates length of short single copy.
6: IRL indicates length of invert repeat region.
7: GN indicates number of genes.
8: IGRL indicates length of intergenic region.
9: GRL indicates length of gene region.
10: Forward slash indicates there is no invert repeat region.
